# Current Concepts on Pathogenic Mechanisms and Histopathology in Cutaneous Lupus Erythematosus

**DOI:** 10.3389/fmed.2022.915828

**Published:** 2022-05-30

**Authors:** Tanja Fetter, Christine Braegelmann, Luka de Vos, Joerg Wenzel

**Affiliations:** Department of Dermatology and Allergy, University Hospital Bonn, Bonn, Germany

**Keywords:** lupus erythematosus, skin inflammation, histology, interface dermatitis, interferon, plasmacytoid dendritic cells, B cells, T cells

## Abstract

Cutaneous lupus erythematosus (CLE) is an interferon (IFN)-driven autoimmune disease that may be limited to the skin or can be associated with systemic lupus erythematosus (SLE). CLE occurs in several morphologic subtypes ranging from isolated, disc-shaped plaques to disseminated skin lesions. The typical histopathologic pattern of skin lesions is named interface dermatitis and characterized by a lymphocytic infiltrate and necroptotic keratinocytes at the dermo-epidermal junction. Other histopathologic patterns primarily involve the dermis or subcutis, depending on the subtype. One critical mechanism in CLE is the chronic reactivation of innate and adaptive immune pathways. An important step in this process is the recognition of endogenous nucleic acids released from dying cells by various pattern recognition receptors (PRRs), including Toll-like receptors (TLRs) and other cytosolic receptors. Crucial cells in CLE pathogenesis comprise plasmacytoid dendritic cells (pDCs) as major producers of type I IFN, T cells exerting cytotoxic effects, and B cells, previously believed to contribute *via* secretion of autoantibodies. However, B cells are increasingly considered to have additional functions, supported by studies finding them to occur in highest numbers in chronic discoid lupus erythematosus (CDLE), a subtype in which autoantibodies are often absent. More precise knowledge of how CLE subtypes differ pathophysiologically may allow a tailored pharmacotherapy in the future, taking into account the specific molecular signature in relation to the morphologic subtype.

## Introduction

Cutaneous lupus erythematosus (CLE) is a heterogeneous autoimmune skin disease that can occur isolated to the skin or with additional systemic manifestation in several organs [systemic lupus erythematosus (SLE)] (1). CLE can be classified based on clinical and histopathologic findings: typical morphological subsets are acute cutaneous (ACLE), subacute cutaneous (SCLE), intermittent cutaneous [ICLE, also termed lupus erythematosus tumidus (LET)], and chronic cutaneous (CCLE) lupus erythematosus (2, 3). CCLE can be further subdivided into chronic discoid lupus erythematosus (CDLE), lupus erythematosus profundus (LEP) and chilblain lupus erythematosus (ChLE), of which CDLE represents the most frequent CCLE subtype (4). ACLE is most commonly associated with SLE—in approximately 80% of cases—whereas localized CDLE only presents with SLE in about 5% of cases (5, 6). CLE subtypes are heterogeneous in their clinical appearance. ACLE and SCLE occur with disseminated maculopapular to gyrated skin lesions, predominantly in sun-exposed skin. In CDLE, scattered disc-like scarring plaques can be found (3). Lupus erythematosus (LE) skin lesions typically feature a histopathologic pattern termed interface dermatitis, defined by the presence of necroptotic keratinocytes and an epitheliotropic cytotoxic lymphocytic infiltrate at the dermo-epidermal junction (7, 8).

The classification of CLE subtypes, however, should not be understood too rigidly as overlaps in clinical and histological appearance are not uncommon. This also supports the assumption of Ackerman, who considers the different CLE subtypes as manifestations of the same pathological process (9). Nevertheless, there is evidence that the individual subtypes differ pathophysiologically, for example, with respect to their cellular composition as recently shown for B cells (10). Not only the molecular differences leading to the different clinical presentations need to be better understood, but also the pathogenic mechanisms of CLE in general: the precise role of involved cell types, the impact of different cytokines described in the disease, and their interaction and regulation in a complex network need further exploration. In the long term, this could help to select a targeted therapy taking the individual molecular profile of a patient into account. A deeper knowledge could also serve to predict the course of the disease, for instance which group of patients with previously isolated CLE lesions will develop SLE.

In this review, we provide an overview of histopathologic patterns observed in different CLE subtypes. We also discuss the current concept of the pathophysiology of CLE. Here, we highlight the cell types and cytokines involved as well as the central mechanisms of chronic reactivation of innate and adaptive immune responses.

## Self-Amplifying Innate and Adaptive Immune Responses as a Hallmark of LE Skin Lesions

In principle, active CLE is characterized by a hyper-activated type I interferon (IFN) pathway, which triggers an inflammatory response against lesional skin (11). This response entails cell destruction, release of proinflammatory mediators and activates immune pathways. The most important step in this proinflammatory vicious cycle is the (re)activation of innate immune pathways by effector mechanisms of the adaptive immune system, leading to a sustained parallel activation of both arms in lesional skin (12, 13).

This vicious cycle can be triggered by provoking factors such as UV light, cigarette smoke and various drugs (14–16). These factors can lead to cellular damage with DNA alterations, such as upregulation of proinflammatory 8-hydroxyguanine (8-OHG) and formation of reactive oxygen species (ROS) (17). Cellular damage can result in apoptosis with release of cellular blebs, which in CLE is initially seen throughout the entire epidermal layer (18). Under physiological conditions, apoptotic cells are engulfed by phagocytes and destroyed within the lysosomes. Moreover, nuclear components are rapidly degraded. However, in CLE, these mechanisms may be defective or of limited efficacy (19). Several factors are assumed to contribute to this deficiency, for example (i) reduced phagocytic activity, (ii) polymorphisms in genes associated with IFN such as IFN-regulatory factor 5 (IRF5) leading to hyper-activation of IFN in response to nucleic acids, (iii) mutations in genes encoding for DNAses such as DNAse I and DNAse III, of which the latter is also known as three prime repair exonuclease 1 (TREX1) (3, 19–24). Interestingly, there is one rare monogenetic variant of ChLE, in which loss of function mutations in TREX1 or activating mutations in the cGAS-STING pathway have been described (25, 26).

These mechanisms lead to secondary necroptosis and thus unwanted release of nuclear components, including nucleic acids and other danger associated molecular patterns (DAMPs) such as high mobility group box 1 protein (HMGB1), reflecting potential autoantigens (27–29). Accumulating nucleic acids can subsequently be recognized by antigen-presenting cells (APCs) and keratinocytes *via* pattern recognition receptors (PRRs) (12). In plasmacytoid dendritic cells (pDCs), Toll-like receptors (TLRs) are considered predominant PRR, which sense nucleic acid motifs as immune complexes bound to autoantibodies (30). In keratinocytes, PRR-recognition is primarily thought to be TLR-independent, although they express TLRs (31–38). Several cytosolic PRR play a role in nucleic acid sensing: (i) the RIG-I-like receptors MDA5 and RIG-I, both enhancing type I IFN expression and (ii) cGAS-STING, also promoting type I IFN expression as well as cell death (39, 40). Moreover, AIM2 (Absent in Melanoma 2) inflammasome activation has been reported (12, 41, 42).

APCs are known to induce the development and clonal expansion of autoantigen-specific B- and T-lymphocytes. Upon repeated autoantigen contact, activated B cells can differentiate into plasma cells to produce specific autoantibodies against nuclear components, and T cells can migrate into lesional tissue to assist in B cell activation and exert cytotoxic effects against keratinocytes, which in turn again leads to the release of endogenous nucleic acids, fueling the self-reinforcing vicious cycle of lesional inflammation (43).

**Figure 1:**
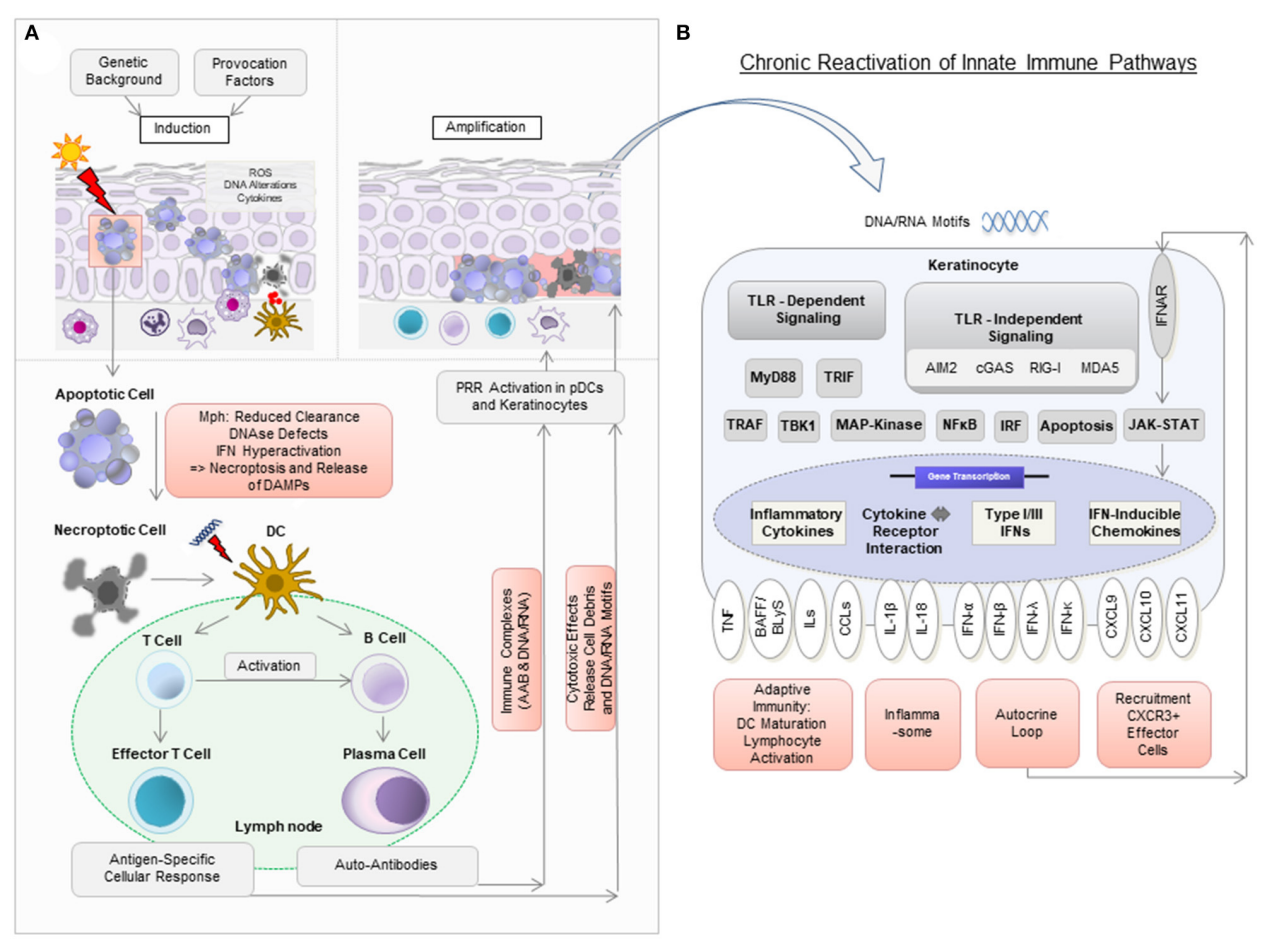
Model of pathogenic mechanisms in cutaneous lupus erythematosus (CLE). **(A)** In a person with a genetic background predisposing to CLE, the exposure to provocation factors such as UV light can induce cellular stress [reactive oxygen species (ROS), DNA alterations, cytokine secretion], apoptosis, and the release of DNA components in so-called “apoptotic blebs” in keratinocytes. Normally, these “blebs” are rapidly degraded and apoptotic cells are removed by macrophages (Mph). In CLE, delayed degradation and clearance leads to secondary, more pro-inflammatory, forms of cell death such as necroptosis, which results in the release of cell debris. Dendritic cells (DC) recognize this debris as potential autoantigens and migrate to nearby lymph nodes to present it to T and B cells. Upon activation, naïve B cells develop into plasma cells to produce autoantibodies (AAB). AAB form immune complexes with nucleic acids and can induce type I IFN production in plasmacytoid dendritic cells (pDCs). T cells mature into CD4+ and CD8+ T cells, with the latter exerting cytotoxic effects against keratinocytes. **(B)** Nucleic acids (DNA and RNA motifs) released from dying cells can be recognized by pattern recognition receptors (PRR) as so-called damage-associated molecular patterns (DAMPs), leading to activation of both Toll-like receptor (TLR)-dependent and TLR-independent inflammatory signaling cascades. In CLE, this leads to increased expression of several cytokines, particularly type I IFN. Type I IFN is known to bind to IFN-α/β receptors on keratinocytes in an autocrine loop and mediates increased expression of proinflammatory chemokines such as CXCL10 via the JAK-STAT pathway. This leads to the recruitment of CXCR3+ cells, which induce keratinocyte cell death, release of cytokines and a chronic reactivation of innate immune pathways.

Neighboring cells can engulf released nucleic acids into the cytosol *via* lipofection—a process known to be mediated by the antimicrobial peptide cathelicidin (44). This enables their subsequent recognition by PRR. Following PRR activation, pDCs and keratinocytes express large amounts of the proinflammatory mediators type I and type III IFNs (especially IFN-κ and IFN-λ) among other cytokines such as several interleukins, Tumor necrosis factor (TNF) and B cell activating factor BAFF, also known as B lymphocyte stimulator BLyS (45–48). IFNs then bind to IFN receptors on keratinocytes in an autocrine loop and induce the expression of IFN-regulated cytokines, most importantly CXCL chemokines (in particular CXCL9, CXCL10, and CXCL11) *via* JAK-STAT signaling (12, 45). CXCL chemokines are known to recruit effector cells expressing the corresponding chemokine receptor CXCR3 (which are CD8+ and CD4+ T cells, pDCs and macrophages) into lesional skin (18). CD8+ T cells can then exert their cytotoxic effect particularly against keratinocytes in the basal epidermal layer, leading to the typical histopathologic pattern of interface dermatitis (Figure 1) (49).

## Characterization of Histopathologic Findings and the Cellular Spectrum in LE Skin Lesions

**Figure 2:**
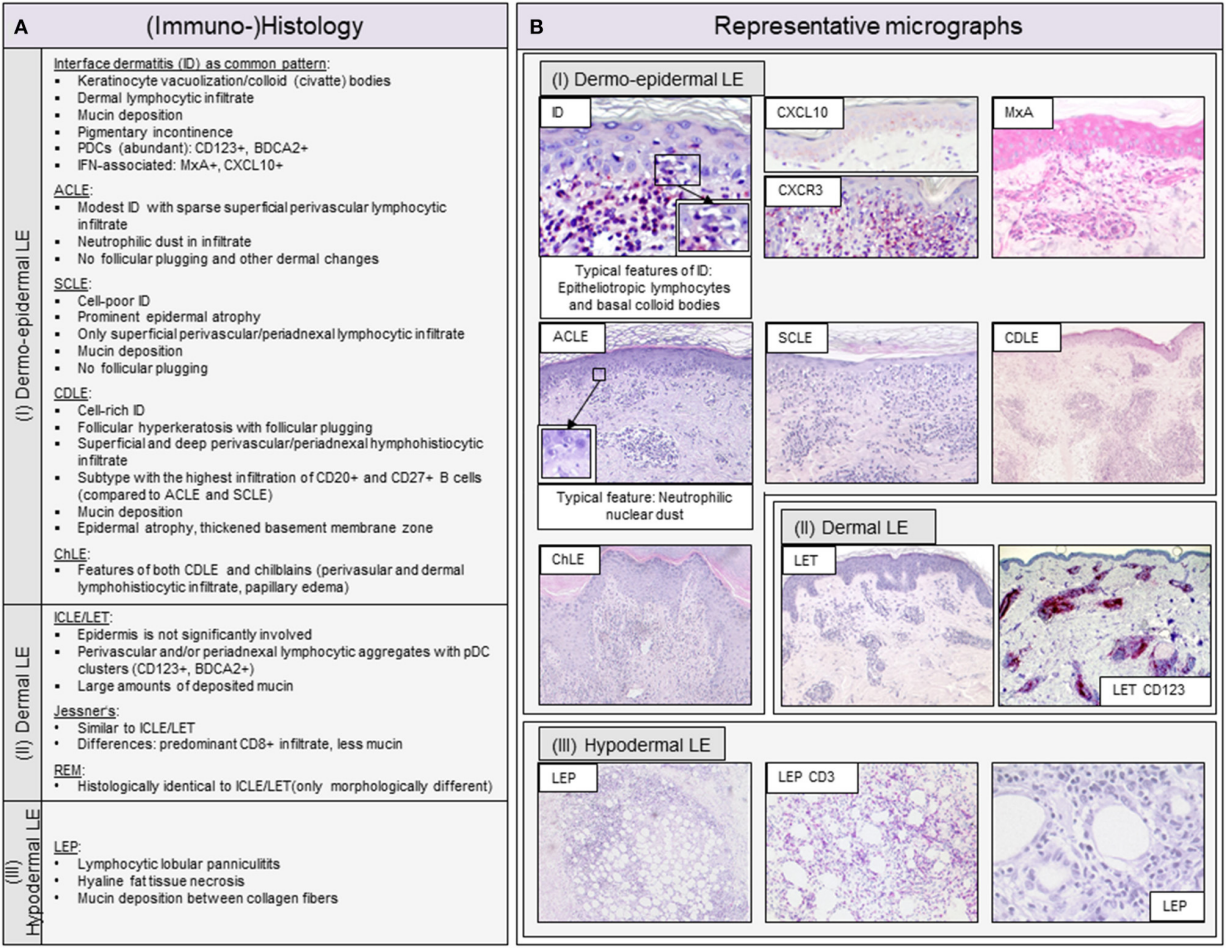
Overview of typical histopathologic patterns observed in different cutaneous lupus erythematosus (CLE) subtypes. **(A)** Typical (immuno-)histologic findings of (I) dermo-epidermal lupus erythematosus (LE), (II) dermal LE and (III) hypodermal LE. Dermo-epidermal LE, presenting as interface dermatitis (ID) includes the morphologic variants acute cutaneous LE (ACLE), subacute cutaneous LE (SCLE) and chronic discoid LE (CDLE) among others. Dermal LE consists of intermittent cutaneous LE (ICLE), also named LE tumidus (LET), Jessner-Kanof lymphocyte infiltrate (Jessner's), and reticular erythematous mucinosis (REM), however, some authors consider Jessner's and REM as separate (only lupus-like) entities. Hypodermal LE includes LE profundus (LEP). ID, interface dermatitis; PDCs, plasmacytoid dendritic cells; IFN, interferon. **(B)** Representative micrographs of different CLE subtypes and selective immunohistochemical features. The typical histopathologic pattern of skin lesions is termed interface dermatitis (ID) and is characterized by epitheliotropic lymphocytes and necroptotic keratinocytes, of which the latter are also called colloid or civatte bodies, at the dermo-epidermal junction. CXCR3+ effector cells are recruited into lesional skin by CXCL10+ expressing keratinocytes. Among these effector cells are CD3+ T lymphocytes, which form the largest immune cell population in LE. The interferon (IFN)-regulated protein MxA reveals a strong expression of IFN in keratinocytes and infiltrating immune cells. ACLE typically features a moderate ID with neutrophilic nuclear dust in the infiltrate. SCLE shows a mild ID with a prominent epidermal atrophy. CDLE features a cell rich ID with a dense perifollicular and perivascular infiltrate and follicular hyperkeratosis and plugging. ICLE/LET presents with a patchy dermal infiltrate and large amounts of deposited mucin. In LEP, a lymphocytic lobular panniculitis can be observed.

The inflammatory cell infiltrate in LE skin lesions varies in composition and distribution depending on the subtype (10, 11, 50). Lipsker has developed a classification of specific histologic findings in CLE based on the primarily affected anatomic structure of the skin (9, 51). He subdivides into (i) dermo-epidermal, (ii) dermal (iii) and hypodermal LE, to which the classic morphological variants can be assigned. The classification of Lipsker is described in more detail with representative micrographs in Figure 2. In the following, we will discuss the main cell types of the innate and adaptive immune system in LE skin lesions.

### Adaptive Immune Cells

The original concept of CLE pathogenesis primarily ascribed a dominant role to the adaptive immune system. This concept emerged primarily from observations in SLE that began about 70 years ago, in which autoantibodies directed against host structures (such as nuclear components) are considered particularly important (52, 53). However, there are CLE patients without a typical autoantibody profile, especially in CDLE (54). Here, the “classical” pathogenic concept is not sufficient to explain the development of the disease. Detailed analyses of skin lesion expression patterns revealed the complex interplay of innate and adaptive immune responses (12).

#### T Cells

CLE is considered a Th1-dominated disease. The pathogenic importance of T cells results from their cytotoxic function, which they exert against structures of the skin, particularly basal keratinocytes (7, 8, 55). Th1 cells promote cellular immune responses as they support cytotoxic T cells and macrophages and produce IFN-γ (49, 56). These cell types represent a central mechanism in the development of the typical histopathologic pattern in all CLE subtypes and contribute to the reactivation of innate immune responses by induction of keratinocyte cell death (49).

#### B Cells

According to the classical concept, B cells are crucial in LE pathogenesis because of their ability to produce autoantibodies against nuclear components. However, this concept could not explain the occurrence of the disease in autoantibody-negative patients (57). Interestingly, some studies reveal a B-cell-rich lesional infiltrate and a strong B cell associated gene signature (e.g., genes encoding B cell activator proteins such as BAFF as well as BAFF receptors) particularly in CLE subtypes lacking autoantibodies such as CDLE (10, 46). Keratinocytes can produce large amounts of BAFF and thus can possibly interact with lesional lymphocytes expressing BAFF receptor (46, 58). In addition, T and B cells appear to gather together in nest-like structures and thus may form a proinflammatory microenvironment (59, 60).

These findings suggest that B cells have other functions besides autoantibody production such as antigen presentation, co-stimulation and cytokine secretion, remaining to be explored in further studies. For instance, an ongoing study investigates the therapeutic effect of the BAFF inhibitor and human monoclonal antibody Belimumab on lesional B lymphocytes in CLE and aims to further characterize these cells (EudraCT 2017-003051-35).

### Innate Immune Cells

#### Plasmacytoid Dendritic Cells

In CLE, Plasmacytoid Dendritic Cells (pDCs) cluster in the dermis to locally produce massive amounts of type I IFN and thus drive the lesional inflammatory process (61, 62). Their direct pathogenic role is underlined by the finding that erasing pDCs in patients with CLE did not only lead to reduction of type I IFN levels, as to be expected, but also reduced disease activity (63). Interestingly, type I IFNs are thought to drive maturation of pDCs (besides many other effects as discussed later) (64), implying a self-amplifying inflammatory process. Immune complexes consisting of nucleic acids and autoantibodies serve as ligands for the activation of pDCs (65). These ligands can be taken up *via* endocytosis with the help of CD32 receptor (66). They are considered to be recognized by PRR through several pathways in parallel: (i) an endosomal way, in which endosomal TLR7 and TLR9 are activated by those ligands and (ii) a cytosolic way, in which the cGAS-STING pathway is activated, both resulting in upregulated type I IFN (and type III IFN) expression (67, 68). Moreover, these pathways most probably interact with each other as the cGAS-STING pathway was shown to dampen the TLR-mediated IFN production in pDCs (67).

#### Neutrophil Granulocytes

In LE skin lesions, neutrophils accumulate primarily during the initial phase of CLE lesion development (69). Neutrophil-released extracellular traps (NETs) are thought to play a pathogenic role in SLE as they can be activated by immune complexes and their degradation is impaired, thus providing a source of potential autoantigens (70). NETs are present in skin lesions of various CLE subtypes and are particularly high in ACLE, CDLE and LEP, suggesting that NETs may be of greater importance in CLE featuring tissue damage and scarring (71).

## Proinflammatory Pathways in CLE

Analysis of gene expression from skin lesions of CLE patients has greatly improved our understanding of immunopathological mechanisms and revealed interesting molecular structures for targeted therapies. A hallmark of all CLE subtypes represents a strongly upregulated IFN pathway as discussed below. Other important signaling pathways include TLR-dependent and TLR-independent (cGAS-STING, RIG-I, MDA5) pathways and their downstream signaling pathways (TRAF, TBK1, NFκB, MAP kinase, IRF), which are known to facilitate the chronic reactivation of innate immune pathways by nucleic acids and other DAMPs. Another well-described pathway in CLE is the JAK-STAT pathway, which is critical in pathogenesis as it is responsible for transmitting IFN signals (12, 72, 73).

## Interferons as Crucial Cytokines in CLE Pathogenesis

The major pathway in CLE pathogenesis is the type I IFN pathway, which has been shown to be upregulated independently of the specific subtype and lead to the suggestion of CLE as an acquired interferonopathy (11, 74). Type I IFNs are of particular importance, with lesional pDCs as major producers (62). Keratinocytes also produce IFNs in response to PRR activation by endogenous nucleic acids (12). Type I IFN-κ has been found to be upregulated in lesional skin and even in clinically healthy skin of LE patients (45). It is probably the major type I IFN produced by keratinocytes (75). To date, the function of IFN-κ is not fully understood. It is assumed to play a role in the development of CLE lesions in clinically healthy skin and to enhance responsiveness to IFN-α and sensitivity to UV light in keratinocytes. Since depletion of IFN-κ was found to abrogate enhanced apoptosis of keratinocytes in response to UV irradiation, IFN-κ may be important in driving apoptotic responses (45).

Type III IFNs have also been detected to be increased in CLE patients with active skin lesions (47). The main representative of this most recently discovered IFN family is IFN-λ (76). Keratinocytes as well as pDCs produce IFN-λ and also express the IFN-λ receptor (47, 77). In cultured keratinocytes, expression of IFN-λ is induced after stimulation with endogenous nucleic acids, following increased expression of IFN-stimulated genes such as CXCL9, CCL3, IL-8 and IL-6 (47). Consistent with previous findings, treatment of lupus-prone mice with IFN-λ led to enhanced levels of proinflammatory cytokines (IL-6, CXCL9, CXCL10, CXCL11) (78). Notably, CXCL10 is of particular importance as it is considered the chemokine that determines the histologic pattern of interface dermatitis (11).

## Molecular Findings from Mouse Models

Lupus prone mouse models enabled insights into molecular mechanisms in CLE. CLE-like skin inflammation can be observed in mice with TREX1^−/−^ knockout and when treated with TLR7 agonists, underscoring the role of innate DAMP signaling in CLE (12, 30, 79). Interestingly, in several studies, TLR9-deficient mice presented with an exacerbation of lupus-like skin lesions, suggesting contradictory effects of TLR7 and TLR9 (80, 81). TLR9 was also shown to suppress the expression of TLR7-dependent autoantibodies, which led to the assumption of cross-regulatory functions (82). Furthermore, mice with an activating JAK1 mutation exhibit CLE-like skin lesions (83), highlighting the importance of the JAK-STAT pathway in this disease.

## Insights into CLE Pathophysiology From Therapeutics

The effectiveness of some therapeutics *in vivo* proves the importance of the respective corresponding targets and signaling pathways in the pathogenesis of CLE. One example is the JAK-STAT pathway: JAK inhibitors have proven to be beneficial in several preclinical studies and case reports in CLE, highlighting the role of the JAK-STAT pathway in the disease (72, 84–87).

However, even when a pathway proves to be particularly relevant, for instance the IFN pathway, it may not be sufficient to block individual components of this pathway, as demonstrated by the limited efficacy of selective anti-IFN-α and anti-IFN-γ antibodies in clinical trials (88–90). It may be necessary to prevent the common downstream effects, e.g., by blocking receptors that transduce signals by several IFNs. Accordingly, a type I IFN receptor antibody proved beneficial on lupus skin lesions (91).

Other treatment options with conflicting results illustrate the complex interplay of immune mechanisms and encourage further analysis of effects that are not yet understood. For example, antimalarials such as hydroxychloroquine are most commonly used in CLE and well tolerated. However, in some cases of CLE and in other autoimmune skin disorders such as dermatomyositis and psoriasis, worsening of skin disease could be observed (92–94). Antimalarials are assumed to inhibit TLR7/TLR9 and cGAS-STING signaling by preventing the binding of nucleic acids to the corresponding receptors (95, 96). They can also inhibit lysosomal activity and autophagy. Autophagy is thought to be involved in antigen presentation leading to adaptive immune responses (97, 98). However, inhibition of endolysosomal activity may also reduce degradation of DAMPs, which could possibly lead to enhanced activation of other (cytosolic) PRRs. Since TLR9 is increasingly considered to actually have anti-inflammatory capacity, concomitant blocking of TLR7 and TLR9 might potentially entail an overall stronger proinflammatory response (99–101). However, this is only one example of paradox effects of therapeutics that require further investigation.

## Conclusion

CLE can be a highly burdensome disease for patients. Fortunately, more insights into CLE pathogenesis have been gained in recent years. A key mechanism is the chronic reactivation of innate immune pathways. *Via* different PRRs, endogenous nucleic acids, released from dying host cells, can be recognized, triggering an IFN- driven inflammatory process that leads to adaptive, especially cytotoxic, immune responses. The findings have led to the development of several targeted therapies that are currently being investigated in clinical trials, partially with promising results. Nevertheless, there is still a need for further therapeutic options, for example for therapy-resistant cases. In order to provide optimal therapy for each individual patient, a deeper understanding of (i) the molecular mechanisms in CLE pathophysiology and (ii) the effects of blocking or modulating a pathway that is part of a complex network is essential. In addition, it is important to determine to what extent the morphological CLE subtypes differ at the molecular level and what leads to the manifestation of a particular subtype. If there are typical molecular features for each subtype, identification of biomarkers would be desirable to reveal the leading mechanisms even in challenging cases with overlapping clinical manifestations. Another task is to better understand the mode of action of therapeutic agents. For instance, it remains to be determined whether and how B-cell-focused strategies such as BAFF inhibitors differ in efficacy in patients frequently featuring autoantibodies (such as ACLE and SCLE) and in patients with particularly high B cell levels in skin lesions lacking autoantibodies (such as CDLE), as they both feature B cell associated processes. A deeper understanding of these mechanisms will hopefully allow stratified or even personalized therapy options for patients in the future.

## Author Contributions

TF and JW performed the literature review and wrote the manuscript. TF, LdV, and JW designed the figures. CB and LdV added intellectual content and critically revised the manuscript. All authors approved the final manuscript for publication.

## Conflict of Interest

The authors have been an advisor and/or received speakers' honoraria or travel expense reimbursements and/or received grants and/or participated in clinical trials of the following companies: CB: Novartis, L'Oreal, GSK, and UCB. JW: GSK, Incyte, Novartis, Medac, Merck/Serono, Roche, Actelion, Pfizer, Spirig, ArrayBio, and Biogen.

## Publisher's Note

All claims expressed in this article are solely those of the authors and do not necessarily represent those of their affiliated organizations, or those of the publisher, the editors and the reviewers. Any product that may be evaluated in this article, or claim that may be made by its manufacturer, is not guaranteed or endorsed by the publisher.
